# Deciphering the Active Compounds and Mechanisms of HSBDF for Treating ALI *via* Integrating Chemical Bioinformatics Analysis

**DOI:** 10.3389/fphar.2022.879268

**Published:** 2022-06-02

**Authors:** Yanru Wang, Xiaojie Jin, Qin Fan, Chenghao Li, Min Zhang, Yongfeng Wang, Qingfeng Wu, Jiawei Li, Xiuzhu Liu, Siyu Wang, Yu Wang, Ling Li, Jia Ling, Chaoxin Li, Qianqian Wang, Yongqi Liu

**Affiliations:** ^1^ Gansu University Key Laboratory for Molecular Medicine and Chinese Medicine Prevention and Treatment of Major Diseases, Gansu University of Chinese Medicine, Lanzhou, China; ^2^ College of Pharmacy, Gansu University of Chinese Medicine, Lanzhou, China; ^3^ Gansu University of Chinese Medicine, Lanzhou, China; ^4^ Institute of Modern Physics, Chinese Academy of Sciences, Lanzhou, China; ^5^ Chronic Disease Research Center, Medical College, Dalian University, Dalian, China; ^6^ Key Laboratory of Dunhuang Medical and Transformation, Ministry of Education of The People’s Republic of China, Lanzhou, China

**Keywords:** Traditional Chinese medicine, Huashi Baidu formula, acute lung injury, inflammation, oxidative stress, apoptosis

## Abstract

The Huashi Baidu Formula (HSBDF), a key Chinese medical drug, has a remarkable clinical efficacy in treating acute lung injury (ALI), and it has been officially approved by the National Medical Products Administration of China for drug clinical trials. Nevertheless, the regulated mechanisms of HSBDF and its active compounds in plasma against ALI were rarely studied. Based on these considerations, the key anti-inflammatory compounds of HSBDF were screened by molecular docking and binding free energy. The key compounds were further identified in plasma by LC/MS. Network pharmacology was employed to identify the potential regulatory mechanism of the key compounds in plasma. Next, the network pharmacological prediction was validated by a series of experimental assays, including CCK-8, EdU staining, test of TNF-α, IL-6, MDA, and T-SOD, and flow cytometry, to identify active compounds. Molecular dynamic simulation and binding interaction patterns were used to evaluate the stability and affinity between active compounds and target. Finally, the active compounds were subjected to predict pharmacokinetic properties. Molecular docking revealed that HSBDF had potential effects of inhibiting inflammation by acting on IL-6R and TNF-α. Piceatannol, emodin, aloe-emodin, rhein, physcion, luteolin, and quercetin were key compounds that may ameliorate ALI, and among which, there were five compounds (emodin, aloe-emodin, rhein, luteolin, and quercetin) in plasma. Network pharmacology results suggested that five key compounds in plasma likely inhibited ALI by regulating inflammation and oxidative damage. Test performed *in vitro* suggested that HSBDF (0.03125 mg/ml), quercetin (1.5625 μM), emodin (3.125 μM), and rhein (1.5625 μM) have anti-inflammatory function against oxidative damage and decrease apoptosis in an inflammatory environment by LPS-stimulation. In addition, active compounds (quercetin, emodin, and rhein) had good development prospects, fine affinity, and stable conformations with the target protein. In summary, this study suggested that HSBDF and its key active components in plasma (quercetin, emodin, and rhein) can decrease levels of pro-inflammatory factors (IL-6 and TNF-α), decrease expression of MDA, increase expression of T-SOD, and decrease cell apoptosis in an inflammatory environment. These data suggest that HSBDF has significant effect on anti-inflammation and anti-oxidative stress and also can decrease cell apoptosis in treating ALI. These findings provided an important strategy for developing new agents and facilitated clinical use of HSBDF against ALI.

## 1 Introduction

Acute lung injury (ALI), an early stage of acute respiratory distress syndrome (ARDS) (Sun et al., 2020), can be caused by various intrapulmonary and extrapulmonary pathogenic factors, such as COVID-19 (Yang et al., 2020). HSBDF was recommended to treat ALI at the later stages of COVID-19 by the National Health Commission of the People’s Republic of China (NHC) (Wang and Qi, 2020) and has been officially approved by the National Medical Products Administration of China for drug clinical trials due to remarkable clinical efficacy. However, its active compounds in plasma and its regulatory mechanisms are not clear yet.

Inflammatory cytokine storm is a characteristic of ALI. Previous studies indicated that interleukin 6 (IL-6) and tumor necrosis factor-α (TNF-α) were key cytokines involved in the pathological process of ALI (Gao et al., 2019). IL-6 and TNF-α were considered critical targets to prevent inflammation in treating ALI.

Molecular docking and network pharmacology are widely used in researching the material basis and mechanism of TCM in the prevention and treatment of disease (Yu R et al., 2020; Yu S et al., 2020; Chen et al., 2021a; Chen et al., 2021b; Chen et al., 2021c; Wang et al., 2021). However, owing to the complexity of TCM and the compounds in plasma remained elusive, it is necessary to make full use of the LC/MS method to conduct the research on active compounds of HSBDF in plasma in treating ALI. Therefore, based on IL-6 and TNF-α, this study aimed to uncover the regulatory mechanisms of HSBDF and its active compounds in plasma against ALI by the integrated strategy of molecular docking, LC/MS, network pharmacology, and *in vitro* approaches, which provide the basic data for further scientific research and clinical use of HSBDF in treating ALI.

## 2 Materials and Methods

### 2.1 Materials and Reagents

*Ephedra sinica* (Shengmahuang, No. 190701), *Agastache rugosus* (Huoxiang, No. 200803003), Gypsum Fibrosum (Shengshigao, No. 201001), *Semen armeniacae amarum* (Kuxingren, No. 202012002), *Rhizoma Pinelliae Preparata* (Fabanxia, No. D2012128), *Magnolia officinalis* (Houpo, No. 202009002), *Atractylodes lancea* (Cangzhu, No. 20210120), *Fructus tsaoko* (Caoguo, No. 20180919), *Poria cocos* (Fuling, JZ2105009), *Astragalus membranaceus* (Shenghuangqi, No. 20210108), *Radix Paeoniae Rubra* (Chishao, No. 2103012), *Lepidium apetalum* (Tinglizi, No. 20210206), *Rheum officinale* (Shengdahuang, No. JZ2012020), and *Glycyrrhiza uralensis* (Gancao, No. 20210201) were obtained from The Affiliated Hospital of Gansu University of Chinese Medicine (Lanzhou, China) at an accurate prescription dose, and their equality control adhered to the specifications and test procedures as described in the Pharmacopoeia of the People’s Republic of China (2020). HSBDF was decocted with water (1:8, w/v) for 60 min. The aqueous solutions were obtained by merging, filtrating, and concentrating to 1.23 g/ml. The extracts were kept at 4°C for preparation of a lyophilized powder for further oral administration to rats and preparation of lyophilized powder.

Methanol and acetonitrile (HPLC pure grade) were purchased from MREDA (MREDA, China), and formic acid of the same grade was provided by Thermo Fisher (Thermo Fisher, Waltham, MA, United States). Ultrapure water was obtained from Wahaha (Wahaha, China) and used throughout the study. Quercetin, luteolin, piceatannol, emodin, rhein, aloe-emodin, and physcion were all purchased from Baoji Herbest Bio-Tech Co., Ltd. (Herbest Bio-Tech, China). The purity of all reference standards was more than 98%. Other reagents of analytical grade were obtained from commercial sources.

LPS, cell counting kit-8, and Click-iT EdU-488 cell proliferation kit were purchased from Solarbio (Beijing, China), Yeasen (Yeasen Biotech, China), and Servicebio (Wuhan, China), respectively. DME/F-12, 1,640, and PBS were obtained from HyClone (HyClone, Logan, UT, United States). Annexin V−FITC/PI apoptosis detection kit was provided by Meilunbio (Dalian, China). MDA (20211202) and T-SOD (20211201) were purchased from Nanjing Jiancheng Bioengineering Institute (Nanjing, China). ELISA kits including TNF-α (2111H27) and IL-6 (2111H35) were purchased from Jiangsu Feiya Biotechnology Co., Ltd. (Jiangsu, China).

### 2.2 Virtual Screening and Pharmacokinetic Prediction

#### 2.2.1 Compounds of Huashi Baidu Formula

The compounds were collected from the traditional Chinese medicine systems pharmacology (TCMSP, https://www.tcmspw.com/tcmsp.php) (Ru et al., 2014) database and Traditional Chinese Medicines Integrated Database (TCMID, http://119.3.41.228: 8000/tcmid/) (Xue et al., 2013). Meanwhile, compounds of each herb were supplemented through a literature search in the PubMed database (https://pubmed.ncbi.nlm.nih.gov/). A total of 1,613 compounds were retrieved for 13 herbs, among which there were *E. sinica* (363), *S. armeniacae amarum* (113), *L. apetalum* (68), *R. paeoniae rubra* (119), *A. rugosus* (94), *M. officinalis* (139), *A. lancea* (49), *F. tsaoko* (59), *Astragalus membranaceus* (87), *Glycyrrhiza uralensis* (280), *R. officinale* (92), *P. cocos* (34), and *R. Pinelliae Preparata* (116). It was worth noting that Gypsum Fibrosum is a mineral drug, and its main composition was CaSO_4_•2H_2_O. Therefore, its mechanism of action was not included in this study.

#### 2.2.2 3D Structures of Protein

The 3D structures of IL-6R (PDB ID: 1P9M) (Ho et al., 2009), TNF-α (PDB ID: 2AZ5) (He et al., 2005), PIK3R1 (PDB ID: 4I6J) (Xing et al., 2013), SRC (PDB ID: 4U5J) (Duan et al., 2014), AKT1 (PDB ID: 4EKL) (Lin et al., 2012), TP53 (PDB ID: 5LAY) (Gollner et al., 2016), and PIK3CA (PDB ID: 7K6M) (Cheng et al., 2021) were downloaded from the RCSB Protein Data Bank (PDB, https://www.rcsb.org/).

#### 2.2.3 Molecular Docking and Binding Free Energy Calculation

3D structures of protein in 2.2.2 were preprocessed by Protein Preparation Wizard. The compounds in 2.2.1 were prepared by the LigPrep module (Sheikh et al., 2018). A total of 1,613 compounds were docked with IL-6R and TNF-α by the Glide standard docking precision method (SP) in Schrődinger 2020-4. The corresponding low-energy conformation was obtained by MMFFs. Next, epik28 (Manchester et al., 2010) used a pH value of 7.0 ± 2.0 to conditionally allocate the ionization state and perform docking calculations.

The binding free energy was used to evaluate the affinity between the ligand and receptor. Lower binding free energy indicates a higher affinity or catalytic activity (Wu et al., 2009). In this study, MM/GBSA in the prime program was used to estimate binding energy (Hou et al., 2011). The formula is: DG bind = E_complex (minimized)—E_ligand (minimized)-E_receptor (minimized).

#### 2.2.4 Molecular Dynamic Simulation and Binding Interaction Pattern Analysis

The initial structures of the protein complex with active compounds (emodin, rhein, and quercetin) were obtained from molecular docking. To further determine the stability of rhein and quercetin with IL-6R and rhein and emodin with the TNF-α complex in a simulated physiological solvent system, we used the Desmond module of Schrödinger to carry out molecular dynamic simulation. The molecular system was solvated with crystallographic water (TIP3P) for a 10-Å buffer region under orthorhombic periodic boundary conditions. The system charges were neutralized by adding Na^+^ and Cl^−^. The molecular system was solvated with crystallographic water (TIP3P) molecules under orthorhombic periodic boundary conditions for a 10 Å buffer region. The system charges were neutralized by adding Na^+^ and Cl^−^. The OPLS_2005 force field was used for energy calculation. After this, an ensemble (NPT) of Nose–Hoover thermostat and barostat was applied to maintain the constant temperature (300°K) and pressure (1 bar) of the systems, respectively. The complex structural dynamic simulation for the best complex was carried out with an NPT ensemble for 50°ns, and the trajectory was set at an interval of 10°ps. The RMSD (root mean square distance) was used to compare the prediction error in disparate models of specific data sets and protein–ligand contacts with the timeline. In addition, the 3D binding interaction pattern of active compounds with protein was visualized by PyMOL.

#### 2.2.5 Pharmacokinetic Prediction of Active Compounds

The parameters of absorption, distribution, metabolism, excretion, and toxicity (ADMET) of emodin, rhein, and quercetin were calculated by using the ProTox-II data platform. In addition, active compounds that conformed to Lipinski’s rule were predicted by the QikProp module of Schrödinger.

### 2.3 LC/MS Analysis

#### 2.3.1 Animals and Drug Administration

A total of six male Sprague-Dawley rats (200 ± 20°g, SPF grade) were purchased from the Laboratory Animal Center of Gansu University of Chinese Medicine (Lanzhou, China) with license number: SCXK (gan) 2020-0001. All animals were housed in the Laboratory Animal Center of Gansu University of Chinese Medicine [animal license number: SCXK (gan) 2020-0009]. The rats were fed standard rodent chow and water *ad libitum* in a house with a 12 h light/dark cycle (temperature and humidity of 23°C ± 2°C and 60% ± 5%, respectively) for 10 days to adapt to the experimental environment. The experimental protocol, approved by the Committee for Ethics in the Laboratory Animal Center of Gansu University of Chinese Medicine (number: 2021-182), was conducted in strict accordance with the ethical principles in animal research.

#### 2.3.2 Instrumentations and Analytical Conditions

The triple quadrupole LC/MS system consisted of an ExionLC (SCIEX, Framingham, MA, United States) and a SCIEX Triple Quad 5,500+ (SCIEX, Framingham, MA, United States) equipped with an ESI source. The chromatographic separation was achieved on a Kinetex C18 column (100 mm × 2.1 mm, 2.6 µm, Kinetex® C18, Phenomenex, United States). The water (containing 0.2% formic acid, solvent A) acetonitrile, and methanol (1:1, v: v, solvent B) system was used as the mobile phase. Analysis was achieved by gradient elution at a flow rate of 0.3 ml/min. The gradient elution program was: 0–2 min, 10% B; 2–5 min, 10%–40% B; 5–15 min, 40%–50% B; 15–30 min, 50%–55% B; 30–50 min, 55%–66% B; 50–63 min, 66%–80% B; and 63–73 min, 80%–100% B. The column and auto-sampler tray temperatures were kept at 30°C and 4°C, respectively. The injection volume was 2 μl. All analytes were quantified using multiple reaction monitoring (MRM) detection in the negative ion mode. The parameters of MRM are shown in Table 1. Other main working parameters were as follows: curtain gas (CUR), ion source gas1 (GS1), and ion source gas2 (GS2) were set at 35, 50, and 50 psi, respectively. IonSpray (IS) voltage, −4,500 V; temperature (TEM), 550°C.

**TABLE 1:** Ions and fragmentations used in the MRM mode for seven compounds.

Compound	MRM fragment	Dwell time (msec)	DP (volts)	DP (volts)	Retention time (min)
Piceatannol	243.1/159	100	−122	−36.7	5.10
	243.1/201.1	100	−122.42	−31.26	
Emodin	269/225	100	−117.21	−39.04	22.39
	269/241	100	−104.02	−39.26	
Aloe-emodin	269.1/240	100	−122.91	−30.76	12.00
	269.1/183.1	100	−122.94	−44.76	
	271.3/239.1	100	−89.09	−51.73	
Rhein	283.1/239	100	−49.87	−24.24	14.77
	283.1/183.1	100	−49.93	−42.03	
	283.1/211.1	100	−50.76	−35.1	
Physcion	283.2/240.1	100	−93.21	−37.75	35.36
	283.2/211.1	100	−68.93	−47	
	283.2/183	100	−66.86	−56	
Luteolin	285.1/133.1	100	−92.87	−45.01	7.62
	285.1/151	100	−118.83	−35.17	
Quercetin	301.1/151	100	−80.95	−27.86	7.77
	301.1/179	100	−75.02	−25.84	

Note: DP, declustering potential; CE, collision energy.

#### 2.3.3 Preparation of Standard Working Solutions, Calibration Standards, Huashi Baidu Formula Extracts, and Plasma Samples

In this study, standard working solutions were made up of mixed standard and single standard. The single standard was made up of quercetin (100 ng/ml). Piceatannol (5,000 ng/ml), emodin (90 ng/ml), rhein (15,000 ng/ml), aloe-emodin (6,000 ng/ml), physcion (150,000 ng/ml), and luteolin (300 ng/ml) were taken 10 µl each to form the mixed standard. Standard working solutions (100 μl) were added to blank plasma (100 μl) to form the calibration standards and stored at 4°C until use.

HSBDF (1.23 g/ml, 20 ml) was filtered, concentrated, dried, and dissolved in 50 ml water (containing 80% analytical-grade methanol) to collect HSBDF extracts (stored at 4°C). Also, the HSBDF extracts were filtered through a 0.22-μm syringe filter when used.

In previous studies, rats fasted overnight with free access to water. The plasma samples (∼0.2 ml) were collected into heparinized microcentrifuge tubes through the caudal vein at 2 h after a single oral administration of HSBDF water decocted. The plasmas were centrifuged at 4,000 rpm for 10 min immediately after collection to obtain plasma. The plasma samples were stored at −80°C until analysis.

The calibration standards and plasma samples were treated with the protein precipitation method using methanol. The procedure was as follows: 100 µl samples were mixed with 800 µl methanol, then vortexed for 5 min, and centrifuged at 13,000 rpm for 10 min. The supernatant was transferred into a clean tube and evaporated with a stream of nitrogen. The residue was dissolved with methanol and was filtered using a 0.22-μm syringe filter to test.

### 2.4 Network Pharmacology Analysis

#### 2.4.1 PPI Analysis

The similarity ensemble approach (SEA) (https://sea.bkslab.org/) (Keiser et al., 2007) was used to predict targets of the key compounds in plasma (emodin, aloe-emodin, rhein, luteolin, and quercetin). The targets were imported into the STRING database (https://string-db.org/) (Szklarczyk et al., 2017), and the organism was set to “Homo sapiens.” The comprehensive score of protein interaction (confidence data >0.9) was used to obtain the PPI information of the target. The major parameter “degree” was used to evaluate its topological features for each node in the interaction network. Based on the degree, bioinformatics (http://www.bioinformatics.com.cn/) was used to visualize the key targets (top 20 targets).

#### 2.4.2 Enrichment

GO and KEGG enrichment was carried out using DAVID (https://david.ncifcrf.gov/) databases. A total of 151 targets of key compounds in plasma were imported using the DAVID database. The select identifier was set to official gene, list type was set to gene list, species was set to “Homo sapiens,” and the threshold was set at *p* < 0.05. Bioinformatics and GOChord of R language were used to construct the GO and pathway-target diagram, respectively.

### 2.5 Test *In Vitro*

#### 2.5.1 Cell Culture

A549 and MLE-12 cells were purchased from Zhong Qiao Xin Zhou Biotechnology Co., Ltd. (Shanghai, China) and maintained at 37°C under 5% CO2 in DME/F-12 and 1,640 medium supplemented with 10% fetal bovine serum, respectively.

HSBDF condensed extract of 1.23 g/ml was lyophilized using a freeze dryer (EYELA, Japan). Finally, 15.38 g of lyophilized powder was obtained (yield, 11.23%). The dry powder was stored in an 80°C refrigerator until use.

#### 2.5.2 Cell Viability Analysis

Cell viability of A549 and MLE-12 cells were measured with the CCK-8 assay after compound intervention. The cells were seeded in 96-well plates at a density of 5 × 10^3^ cells/well in 200 μl of culture media and grown for 4 h. Second, they were treated with different concentrations of LPS, quercetin, luteolin, emodin, rhein, aloe-emodin, and HSBDF. Subsequently, 10 μl of CCK-8 solution was added to each well, and the plates were incubated for 1 h. The optical density of each well was determined at 450 nm using a microplate reader (Bio-Rad, Hercules, CA, United States).

#### 2.5.3 EdU Proliferation Analysis

EdU proliferation kit was employed for the determination of DNA synthetic capacity in proliferating cells. A549 cells were cultured in 96-well plates and grown for 4 h. After being treated for 24 h with compounds, the cells were strictly conducted following the instructions of the kit. Live cell imaging system (OLYMPUS, Japan, 20×) and ImageJ software were employed for calculating the number of EdU-positive cells (green cells). The percentage of EdU-positive cells to total Hoechst 33342-positive cells (blue cells) was represented as the EdU-positive rate.

#### 2.5.4 Enzyme-Linked Immunosorbent Assay, MDA, and T-SOD Analysis

The A549 cells culture medium was gathered and centrifuged (3,000 r/min×20 min) at 4°C. The supernatant liquid was taken out for detection of MDA, T-SOD, TNF-α, and IL-6. Enzyme-Linked Immunosorbent Assay (ELISA) (TNF-α and IL-6), MDA, and T-SOD steps were followed as per the instructions of the reagent kit.

#### 2.5.5 Flow Cytometry Analysis

FITC-conjugated annexin V was used to detect apoptosis. A549 cells were cultured in six-well plates (1 × 10^5^ cells/well) for 4 h before treatment. After adding LPS, quercetin, emodin, rhein, and HSBDF, the cells were incubated for 24 h. Next, the cells were gathered and stained using the annexin V−FITC/PI apoptosis detection kit following the manufacturer’s instructions. The resulting fluorescence signal was detected by flow cytometry (BD, FACSCelesta).

#### 2.5.6 Statistical Analysis

Analyses were performed using GraphPad Prism 6 software. The results are expressed as mean ± standard deviation (SD) of at least three independent experiments. The statistical differences between the two groups were compared by Student’s t-test. *p* < 0.05 was considered statistically significant.

## 3 Results and Discussion

### 3.1 Analysis of Key Anti-Inflammatory Compounds of Huashi Baidu Formula

#### 3.1.1 Huashi Baidu Formula Had Potential Effects to Inhibit Inflammation

To explore the anti-inflammatory effect of HSBDF, 1,613 compounds were docked with IL-6R and TNF-α by Schrődinger. The compound with a docking score of ≤ −5 Kcal/mol was known as the potentially effective compound (Hsin et al., 2013). It can be seen that the total number of compounds with potential inhibitory activities was IL-6R (96) and TNF-α (111) as shown in Table 2, indicating that the formula had potential effects on inhibiting inflammation by acting on IL-6R and TNF-α.

**TABLE 2:** Number statistics of molecular docking.

No	Latin name of the herb	Chinese name of the herb	Number of compounds with docking score ≤−5 Kcal/mol	
			IL-6R	TNF-α
1	*E. sinica*	Shengmahuang	17	25
2	*S. armeniacae amarum*	Kuxingren	2	5
3	*L. apetalum*	Tinglizi	5	2
4	*R. Paeoniae Rubra*	Chishao	9	7
5	*A. rugosus*	Huoxiang	5	5
6	*M. officinalis*	Houpo	1	8
7	*A. lancea*	Cangzhu	0	1
8	*F. tsaoko*	Caoguo	7	6
9	*A. membranaceus*	Shenghuangqi	13	6
10	*G. uralensis*	Gancao	30	41
11	*R. officinale*	Shengdahuang	19	16
12	*P. cocos*	Fuling	0	0
13	*R. Pinelliae Preparata*	Fabanxia	8	11
Total			96	111

Note: The total number in the table is the result after weight removal.

In terms of a single herb, it was found that *Glycyrrhiza uralensis*, *Ephedra sinica*, and *Rheum officinale* were the top three docking herbs with two targets, respectively. Previous studies also demonstrated that Glycyrrhiza polysaccharide (50 mg/kg) had a certain therapeutic effect on lung inflammation and oxidative damage in COPD mice (Wu et al., 2020); *Ephedra sinica* water plays an important role in distillates in arthritic rats (Yeom et al., 2006); *Rheum officinale* ethanol extract can effectively improve the pulmonary inflammation in mice infected with mycoplasma pneumonia (Gao et al., 2021). These results suggested that *Glycyrrhiza uralensis*, *Ephedra sinica*, and *Rheum officinale* were key herbs to inhibit inflammation, which was of great significance to ameliorate ALI.

#### 3.1.2 Piceatannol, Emodin, Aloe-Emodin, Rhein, Physcion, Luteolin, and Quercetin Were Key Compounds

To determine anti-inflammatory key compounds, we selected the compounds, which conformed to Lipinski’s rules (MW < 500, H-bond donor<5, H-bond acceptor<10, log P (O/W) < 5, and rotor ≤10) and docking score ≤−5 Kcal/mol, to analyze further. Based on the Pharmacopoeia of the People’s Republic of China (2020) and the literature of every herb’s main components, seven key compounds were selected from the top 50 compounds with high docking scores (Table 3). These compounds’ binding free energy was further calculated in Schrődinger.

**TABLE 3:** Information of the representative compounds.

Target	Constituent	Herb	Docking score (Kcal/mol)	Binding free energy (Kcal/mol)
IL-6R	Aloe-emodin	*R. officinale*	−5.73	−38.50
	Physcion	*R. officinale*	−5.43	−39.68
	Rhein	*R. officinale*	−5.31	−23.75
	Luteolin	*E. sinica*	−5.12	−36.29
	Quercetin	*E. sinica*, *L. apetalum*, *A. rugosus*, *F. tsaoko*, *A. membranaceus*, and *G. uralensis*	−5.08	−39.35
TNF-α	Aloe-emodin	*R. officinale*	−5.57	−36.60
	Piceatannol	*R. officinale*	−5.38	−37.68
	Emodin	*R. officinale*	−5.36	−36.16
	Rhein	*R. officinale*	−5.28	−29.87
	Physcion	*R. officinale*	−5.28	−38.41
	Luteolin	*E. sinica*	−5.24	−38.38

The results indicated that piceatannol, emodin, aloe-emodin, rhein, physcion, luteolin, and quercetin had potential effects on inhibiting inflammation. Therefore, these compounds were considered key compounds to explore further. Also, we found that these compounds were mainly distributed in *Glycyrrhiza uralensis*, *E. sinica*, and *R. officinale*, which further provides support that *Glycyrrhiza uralensis*, *E. sinica*, and *R. officinale* were key herbs to treat ALI.

### 3.2 Key Compounds in Plasma by LC/MS

**FIGURE 1:**
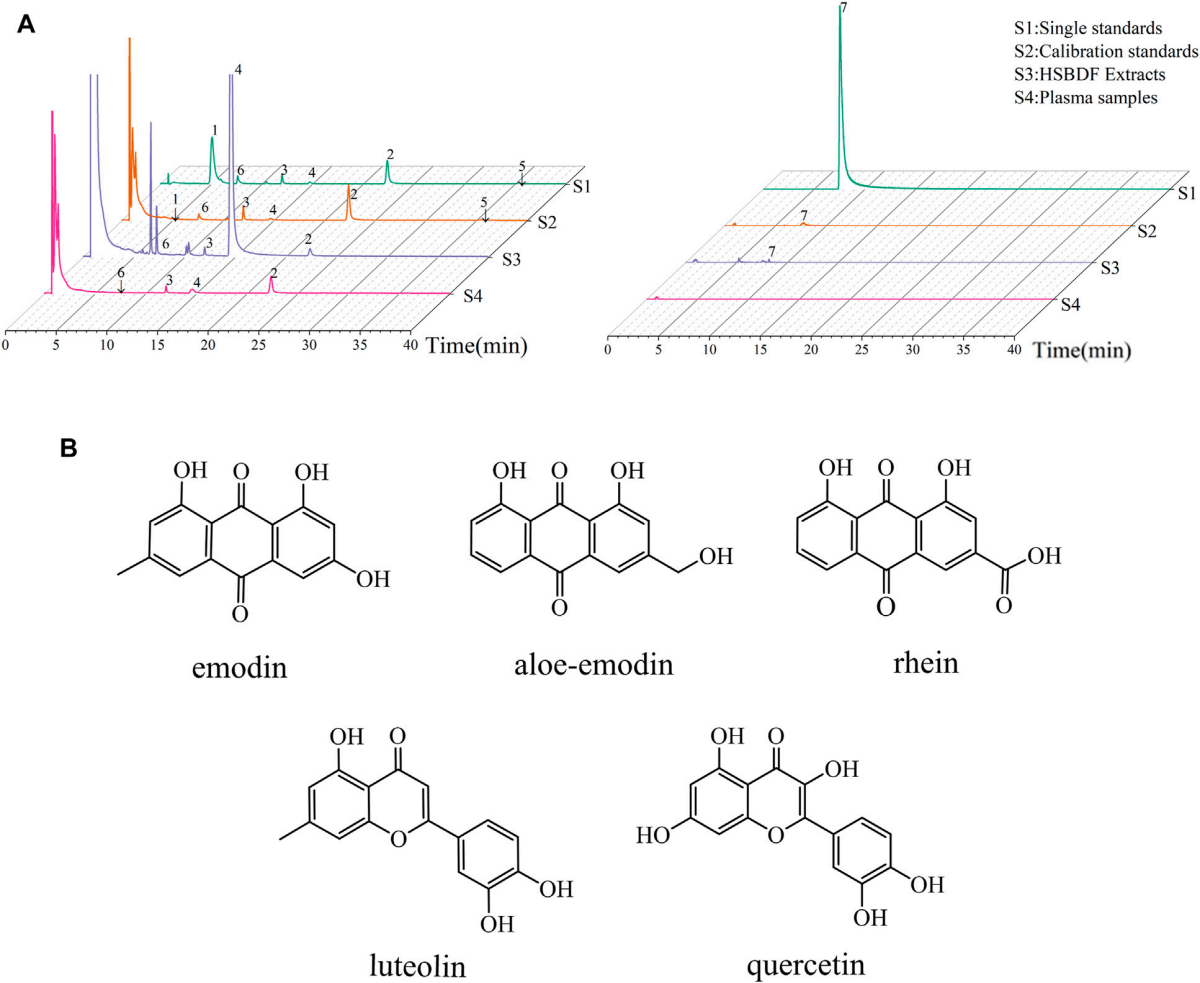
Key compounds analysis by LC/MS. **(A)** MRM chromatograms of key compounds (1–7 represent piceatannol, emodin, aloe-emodin, rhein, physcion, luteolin, and quercetin, respectively); **(B)** structure of key compounds in plasma.

In order to further analyze whether the seven key compounds (piceatannol, emodin, aloe-emodin, rhein, physcion, luteolin, and quercetin) were present in plasma, LC/MS was used. The results of preliminary experiments showed that the number of chromatographic peaks and intensity of peaks were the most strongly detected in 2 h plasma after HSBDF administration. Therefore, 2 h was selected to collect plasma after drug administration. Ion chromatograms of standards (S1) of key compounds, calibration standards (S2), HSBDF extracts (S3), and plasma sample (S4) are displayed in Figure 1A. LC/MS analysis showed that a total of five compounds (emodin, aloe-emodin, rhein, luteolin, and quercetin) were identified in HSBDF extracts; four compounds (emodin, aloe-emodin, rhein, and luteolin) were identified in the plasma sample at 2 h, considering that quercetin was rapidly hydrolyzed after oral administration (Jiang and Hu, 2012).

Therefore, LC/MS analysis showed that there were five key compounds in plasma of HSBDF, including emodin, aloe-emodin, rhein, luteolin, and quercetin. The structures of these compounds are shown in Figure 1B.

### 3.3 Network Pharmacology of Key Compounds in Plasma

#### 3.3.1 PPI Analysis and Molecular Docking Verification

**FIGURE 2:**
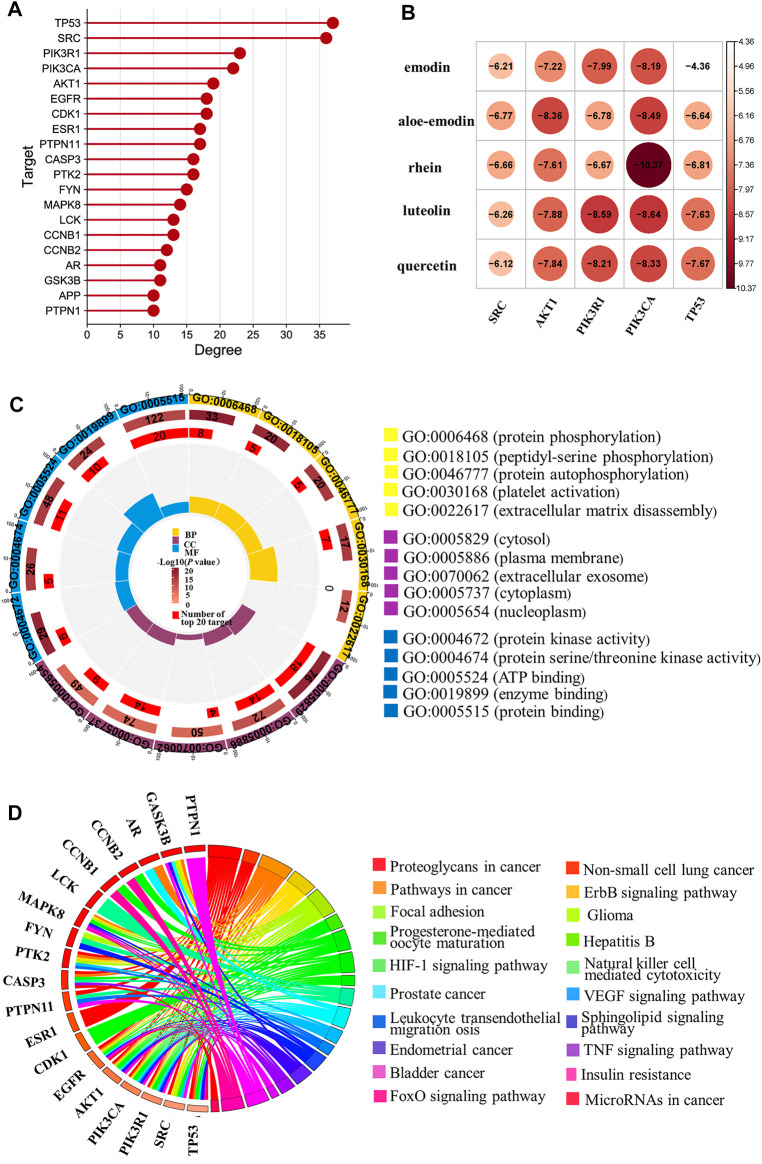
Network pharmacology analysis of key compounds in plasma. **(A)** Top 20 targets of PPI; **(B)** molecular docking results of the key compounds in plasma with top five targets of PPI; **(C)** GO enrichment of the key compounds in plasma; **(D)** pathway-target diagram of key compounds in plasma.

It was well known that network pharmacology contributes to exploring the potential mechanism of TCM in prevention and treatment of disease (Li et al., 2021). Therefore, in order to analyze the mechanism of key compounds in plasma, top 20 in 151 (confidence data >0.9) targets of PPI are listed in Figure 2A. The result showed that the top 20 targets of degree were TP53, SRC, PIK3R1, PIK3CA, AKT1, EGFR, CDK1, ESR1, PTPN11, CASP3, PTK2, FYN, MAPK8, LCK, CCNB1, CCNB2, AR, GSK3B, APP, and PTPN1.

In order to further validate the prediction of network pharmacology, five nodes, namely, TP53, SRC, PIK3R1, PIK3CA, and AKT1 with the highest degree were taken as examples to be analyzed. Their crystal structure was subjected and docked with corresponding compounds. Finally, constituent with docking score ≤ −5 (Kcal/mol) was selected to analyze. The total proportion of TP53 (5/5), SRC (5/5), PIK3R1 (5/5), PIK3CA (5/5), and AKT1 (4/5) was 96%, and the results are shown in Figure 2B. The results of molecular docking showed that the prediction accuracy was high, which verified the result of PPI.

It was reported that SRC could inhibit TLR4-induced inflammatory cytokines and promote anti-inflammatory cytokine IL-10, which plays an important role in the treatment of acute and chronic inflammation (Li et al., 2019). The pharmacological study suggested that *G. baccata* extract can upregulate *AKT1* gene expressions to protect the cells against oxidative damage and inflammation (Martínez et al., 2021). Moreover, the studies also suggested that TP53, PIK3R1, and PIK3CA were vital for inflammation (Eyileten et al., 2018; Busbee et al., 2021; Reske et al., 2021). Accordingly, the results indicate that emodin, aloe-emodin, rhein, luteolin, and quercetin may play an important role in inhibiting anti-inflammatory and anti-oxidative damage.

#### 3.3.2 GO and KEGG Enrichment Analysis

To explore the regulatory mechanisms of key compounds in plasma, a total of 267 terms were enriched (*p* < 0.01), and the top five of GO enrichment were visualized, as shown in Figure 2C. Among which, biological processes (BP) include protein phosphorylation, peptidyl-serine phosphorylation, and protein autophosphorylation, etc; cell composition (CC) includes cytosol, plasma membrane, extracellular exosome, cytoplasm, and nucleoplasm; and molecular function (MF) includes protein kinase activity, protein serine/threonine kinase activity, ATP binding, and so on.

Moreover, we had used 151 targets to enrich 107 pathways (*p* < 0.05), and the top 20 targets and top 20 pathways are shown in Figure 2D. It can be seen that the HIF-1 signaling pathway, the ErbB signaling pathway, the TNF signaling pathway, and so on may be the key regulated pathways of emodin, aloe-emodin, rhein, luteolin, and quercetin. Also, studies have shown that the HIF-1α signaling pathway was related to inflammation, and arginine can regulate HIF-1α to suppress the inflammatory response (Chen et al., 2020). The Er7bB signaling pathway was associated with oxidative stress, and it was reported that the ErbB4 system of the heart was activated at early stages of chronic heart failure to enhance the cardiomyocyte resistance to oxidative stress (Shchendrigina et al., 2020). The TNF signaling pathways and inflammatory pathways can suppress inflammation (Liang et al., 2021).

Network pharmacology results suggested that emodin, aloe-emodin, rhein, luteolin, and quercetin likely treated ALI by regulating the biological function and pathways related to anti-inflammatory and anti-oxidative damage.

### 3.4 Test *In Vitro* of Key Compounds in Plasma

#### 3.4.1 Inflammatory Model With LPS

**FIGURE 3:**
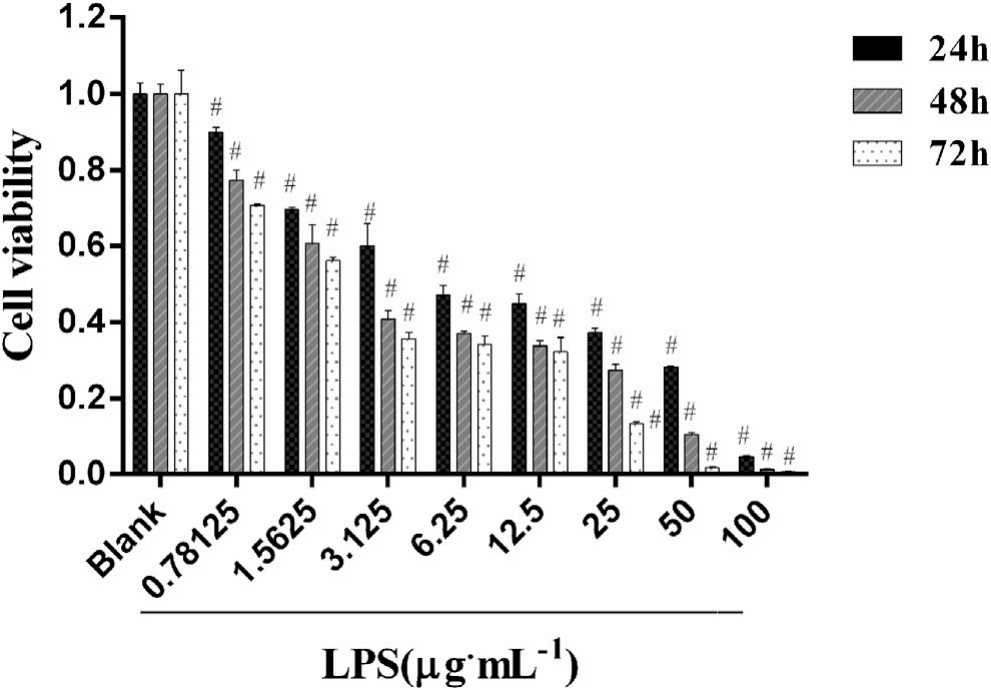
Cell Viability after different treatments of LPS (*p < 0.05, ^#^ *p* < 0.01 versus blank).

A549 is a human alveolar epithelial cell line. It is often used to research lung diseases, such as ARDS (Lin et al., 2020), ALI (Geiser et al., 2001; Zhao et al., 2021). Therefore, we used A549 to further study ALI. To determine the optimal concentration and time of LPS, cell counting kit-8 (CCK-8) was used to detect the viability of A549 cells in this study. As shown in Figure 3, LPS markedly decreased the cell viability of A549 cells in a dose- and time-dependent manner. The cell viability was reduced by approximately 50% when A549 was subjected to 6.25 μg/ml of LPS compared with blank (*p* < 0.01) in 24 h, indicating that 6.25 μg/ml of LPS is deleterious to the epithelial cell. LPS of 12.5, 25, 50, or 100 μg/ml decreased cell viability more prominently than 6.25 μg/ml LPS did, which demonstrated that LPS induced cell viability loss in a dose-dependent manner and that the lowest effective injury dose of 24 h LPS treatment was 6.25 μg/ml (Liu et al., 2021). Consequently, further experiments were performed with 6.25 μg/ml of LPS at 24 h.

#### 3.4.2 Cell Viability of the Huashi Baidu Formula and Key Compounds in Plasma

**FIGURE 4:**
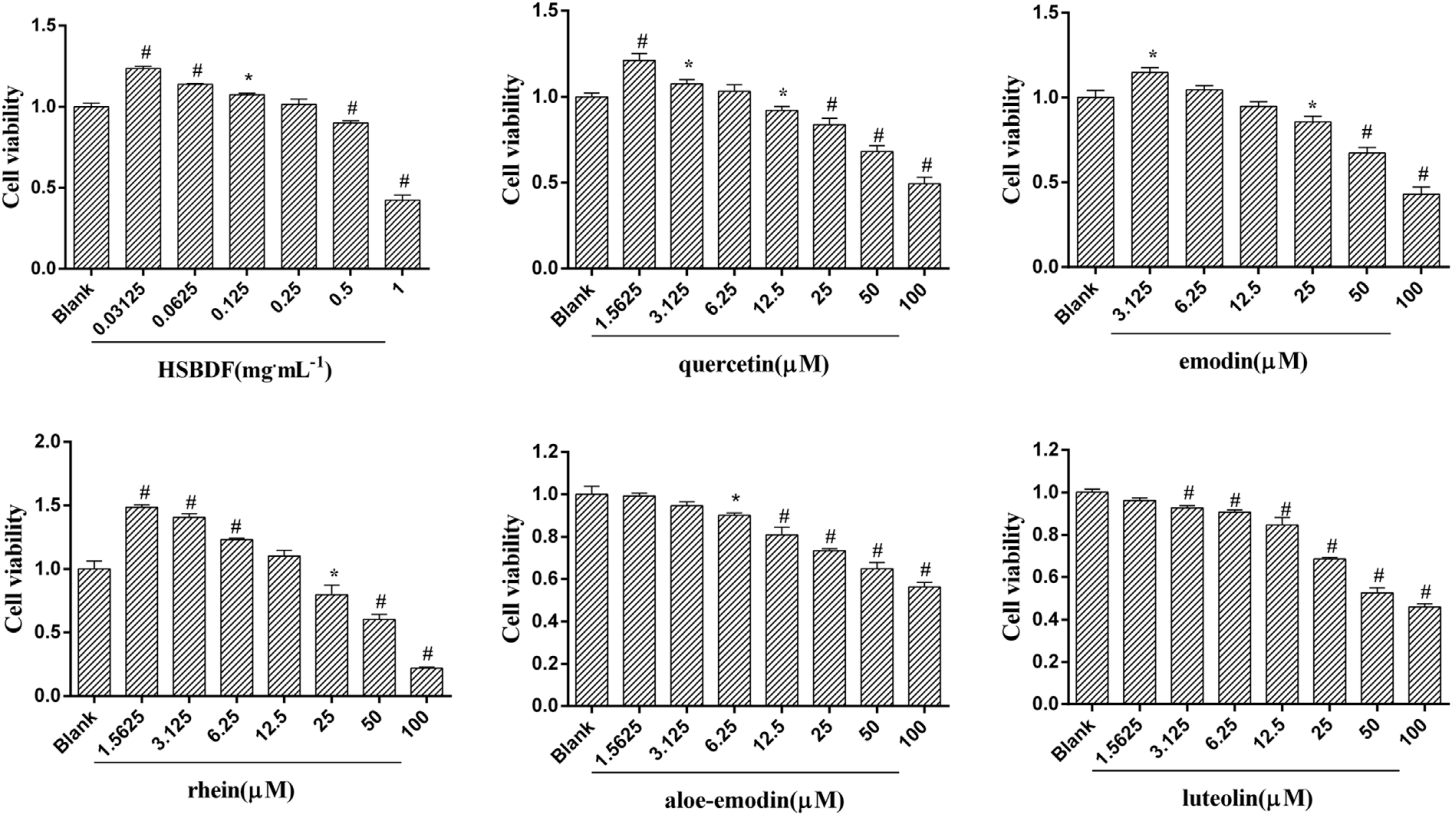
Cell viability after treatments of HSBDF and key compounds in plasma. (cell viability was detected by CCK-8; *p < 0.05, ^#^ *p* < 0.01 versus blank).

In order to determine the effect of HSBDF and key compounds in plasma on the viability of A549 cells, CCK-8 was used to detect the viability of cells treated with different concentrations of drug for 24 h (Figure 4). The results showed that HSBDF and key compounds in plasma can increase the cytotoxicity of A549 cells in a dose-dependent manner. Moreover, we found that A549 cell proliferation was markedly promoted with low concentration of quercetin (1.5625 μM), emodin (3.125 μM), rhein (1.5625 μM), and HSBDF (0.03125 mg/ml), whereas aloe-emodin and luteolin did not. The cell viability was further tested for normal epithelial cells (MLE12) after treating with low concentration of quercetin, emodin, and rhein. (Supplementary Figure S1). We found that the viability of lung epithelial cells, such as A549 and MLE-12, can be increased by these key compounds.

#### 3.4.3 Huashi Baidu Formula and Active Compounds Promote A549 Cell Proliferation in an Inflammatory Environment

**FIGURE 5:**
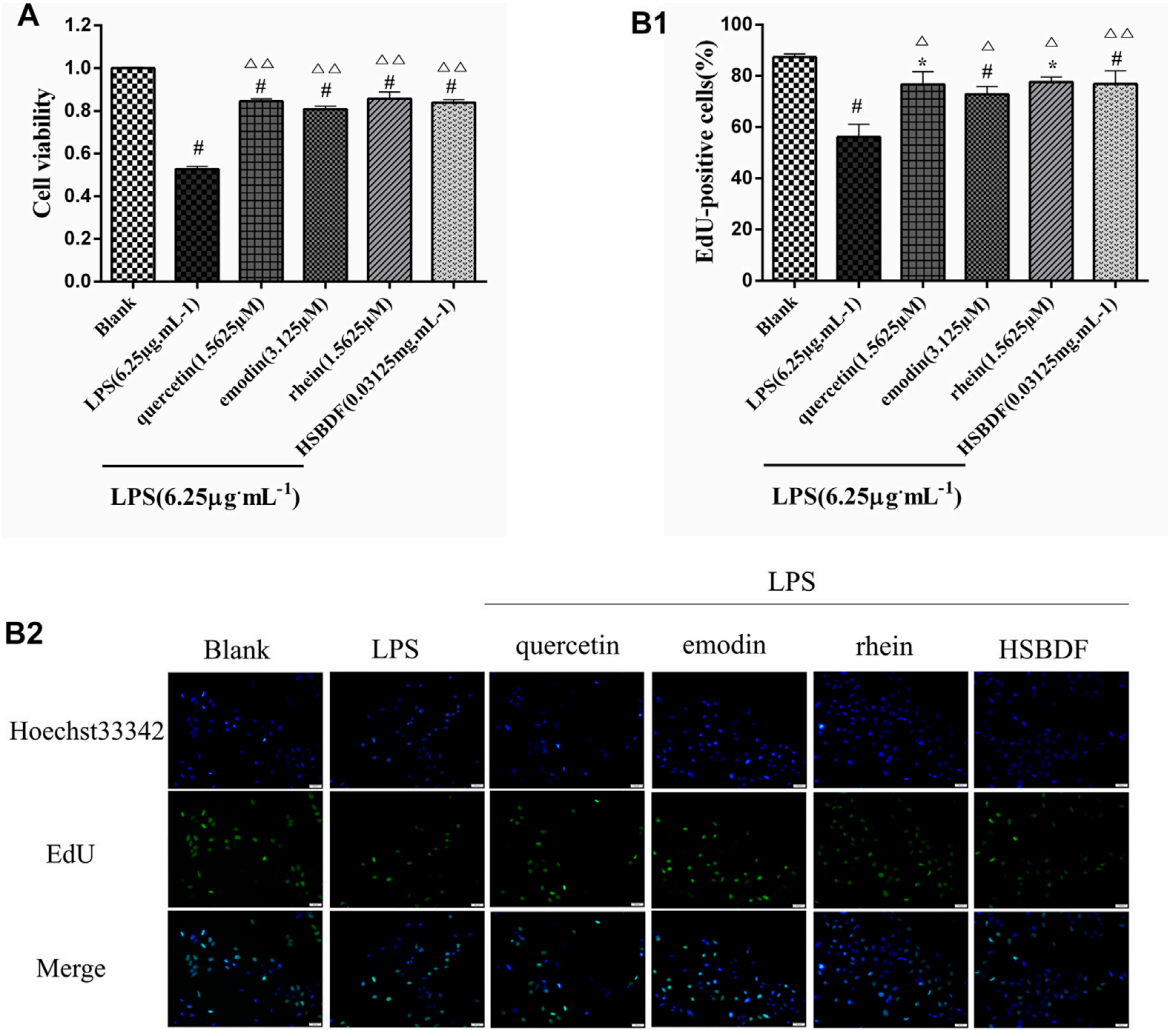
Effect of HSBDF and active compounds treatment on cell proliferation in LPS-stimulated A549 cells. **(A)** Cell proliferation test by CCK-8; **(B1,B2)** DNA synthesis activity in cells examined by EdU staining (magnification, x20) (*p < 0.05, ^#^ *p* < 0.01 versus blank; △*p* < 0.05, △△*p* < 0.01 versus LPS).

To further evaluate the effects of HSBDF, quercetin, emodin, and rhein on A549 cells grown in an inflammatory environment, the EdU proliferation kit and cck-8 were used to detect proliferating cells and the viability of cells, respectively. As shown in Figure 5A, we found that inflammation stimulated by LPS inhibited the proliferation of A549 cells, whereas inhibition of proliferation was decreased by quercetin (1.5625 μM), emodin (3.125 μM), rhein (1.5625 μM), and HSBDF (0.03125 mg/ml). The stimulative proliferation of quercetin (1.5625 μM), emodin (3.125 μM), rhein (1.5625 μM), and HSBDF (0.03125 mg/ml) on A549 cells cultured in an inflammatory environment was also confirmed by EdU proliferation staining (Figures 5B1,B2). These results demonstrate that quercetin, emodin, rhein, and HSBDF were effective in inhibiting inflammation. Therefore, HSBDF (0.03125 mg/ml), quercetin (1.5625 μM), emodin (3.125 μM), and rhein (1.5625 μM) were active compounds for further experiments.

#### 3.4.4 Huashi Baidu Formula and Active Compounds Decrease Expression of TNF-α and IL-6, Decrease Expression of MDA, and Increase Expression of T-SOD in A549 Cells in an Inflammatory Environment

**FIGURE 6:**
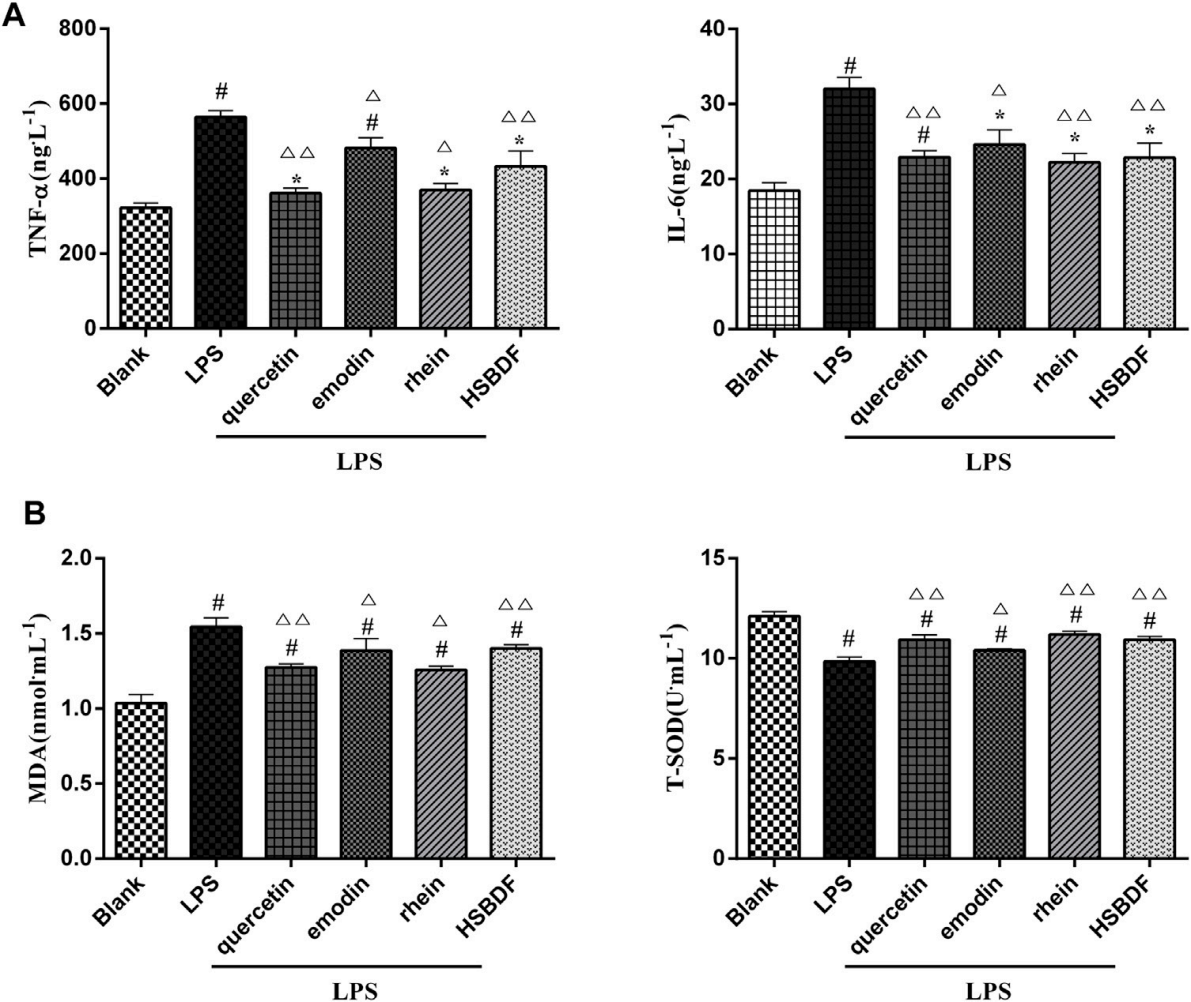
**(A)** Effects of HSBDF and active compound treatment on the expression levels of inflammatory cytokine (IL-6 and TNF-α) in LPS-stimulated A549 cells by the ELISA test. **(B)** Effects of HSBDF and active compound treatment on oxidative stress (MDA and T-SOD) in LPS-stimulated A549 cells (*p < 0.05, ^#^ *p* < 0.01 versus blank; ^△^ *p* < 0.05, ^△△^ *p* < 0.01 versus LPS).

To determine the effect of HSBDF and active compounds on the expression of inflammatory cytokine, ELISA was used to measure the expression levels of IL-6 and TNF-α in the cell supernatant liquid. The elevated expressions of TNF-α and IL-6 induced by LPS in A549 cells were inhibited by quercetin (1.5625 μM), emodin (3.125 μM), rhein (1.5625 μM), and HSBDF (0.03125 mg/ml) (Figure 6A).

Simultaneously, the anti-oxidative stress of HSBDF and active compounds was further researched in an inflammatory environment. We found that the oxidative stress injury was weakened after treatment of HSBDF and active compounds. The SOD was enhanced significantly by active compounds, while the MDA was decreased (Figure 6B).

These results further verify that network pharmacology results indicate that emodin, rhein, and quercetin have the function of anti-inflammation and act against oxidative damage.

#### 3.4.5 Huashi Baidu Formula and Active Compounds Inhibit A549 Cell Apoptosis in an Inflammatory Environment

**FIGURE 7:**
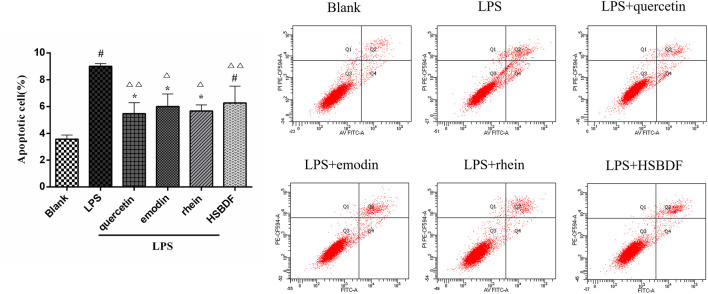
Effects of HSBDF and active compound treatment on apoptosis in LPS-stimulated A549 cells (*p < 0.05, ^#^ *p* < 0.01 versus blank; ^△^ *p* < 0.05, ^△△^ *p* < 0.01 versus LPS).

LPS has been known to cause alveolar epithelial cell apoptosis that characterizes ALI (Hou et al., 2021). Therefore, the anti-apoptosis of HSBDF and active compounds was further investigated in an inflammatory environment. We investigated the effects of drugs on the apoptosis of A549 cells in an inflammatory environment by flow cytometry. We found that LPS increased A549 cell apoptosis. The apoptosis was significantly decreased after the intervention of quercetin, emodin, rhein, and HSBDF in an inflammatory environment (Figure 7).

## 4 Molecular Dynamic Simulation and Binding Interaction Pattern of the Complex of Active Compounds and Protein

**FIGURE 8:**
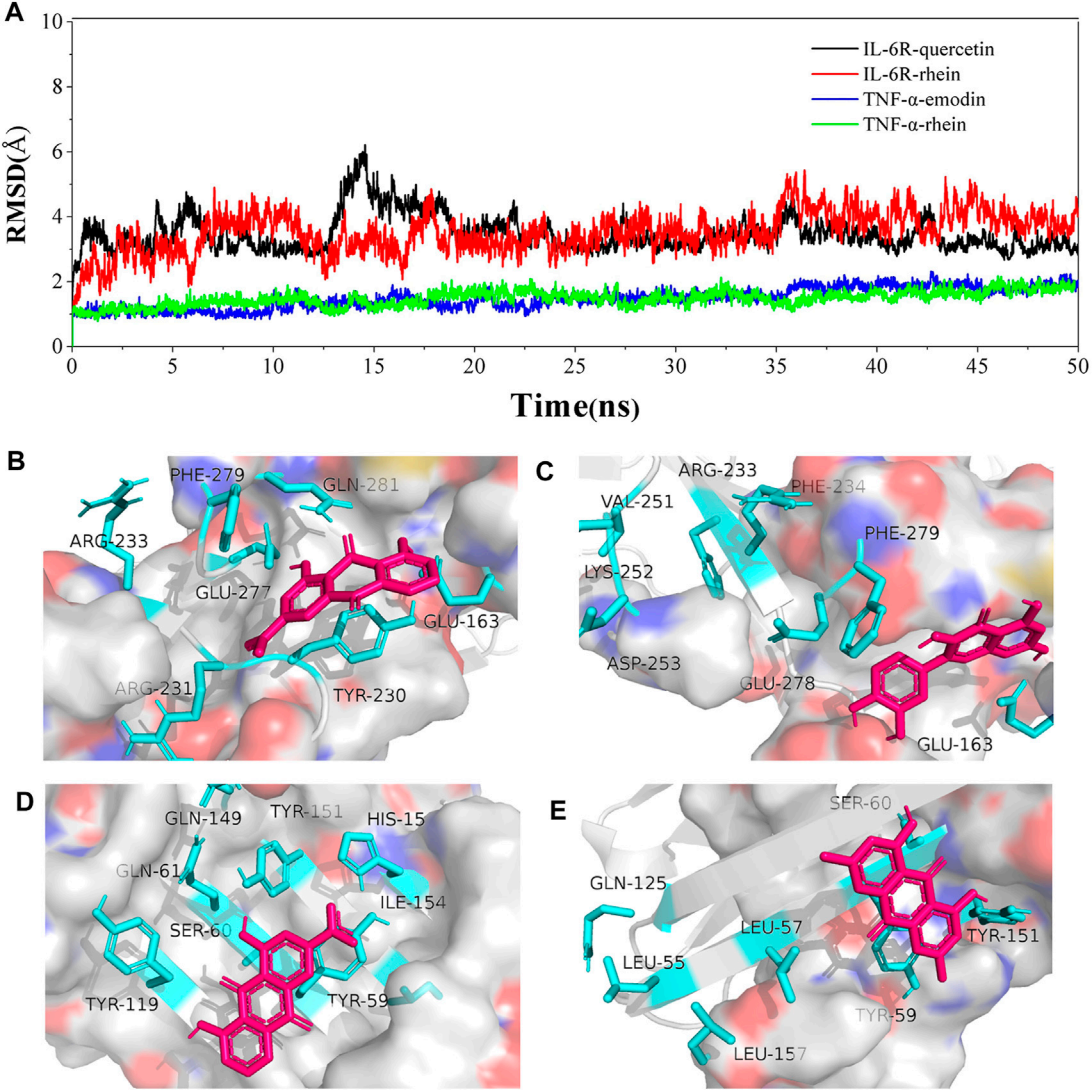
Molecular dynamics simulation study. **(A)** RMSD of the active compound–protein complex in 50 ns, which is made up of a number of α-carbon (Cα) atoms, throughout the simulations; **(B,C)** Binding interaction patterns of rhein and quercetin with IL-6R; **(D,E)** Binding interaction patterns of rhein and emodin with TNF-α.

To further confirm the molecular docking insights and analyze the conformation stability of active compounds and target proteins, molecular dynamic simulations were conducted for rhein and quercetin with IL-6R and rhein and emodin with the TNF-α complex in 50 ns by the Desmond module. The root mean square deviation (RMSD) plot in Figure 8A showed that the fluctuation values of RMSD of both systems were stable within a certain range by simulating 50 ns, which reveals that the simulation was well equilibrated during 50 ns and the ligand did not undergo a remarkable conformational change.

The interactions of active compounds and target proteins were further elaborated based on the hydrogen bonds, hydrophobic, ionic, and water bridges during 50-ns simulation. As shown in Figure 8A and Supplementary Figure S2, the interaction between rhein and IL-6R’s residues (Figure 8B), such as GLU277, PHE279, GLN281, ARG233, ARG231, GLU163, and TYR230, was a stable key, for example, the H-bond and hydrophobic interaction between rhein and GLU277 and PHE29, respectively (Figure 8C). The interaction between quercetin and IL-6R’s residues, such as ARG233, PHE234, VAL251, LYS252, ASP253, GLU278, PHE279, and GLU163, was a stable key, for example, the hydrophobic interaction betweenARG233 and quercetin. TYR59, TYR151, HIS15, GLY149, TYR119, GLN61, ILE154, and SER60 were key interacting residues of TNF-α, among which, TYR59 and TYR151 formed a stable hydrophobic and H-bond interaction with rhein, respectively (Figure 8D). TYR59, GLN125, TYR151, LEU157, LEU55, LEU57, and SER60 were key interacting residues of TNF-α, which mainly formed H-bond and hydrophobic and ionic interaction with emodin (Figure 8E).

These results suggested that binding of ligand molecules (rhein, quercetin, and emodin) stabilizes the protein structure without noticeable structural changes in native conformation. That is the conformation of the complex was stable, and the residues are derived from stable complexes.

## 5 Pharmacokinetic Properties of Active Compounds

The ADMET property prediction is fundamental for the selection of the most promising compounds for further development. The active compounds (quercetin, emodin, and rhein) were subjected to predictions of pharmacokinetic properties using ProTox-II and QikProp. As shown in Table 4, active compounds conformed to RO5 and QPlogBB, and CNS showed that a low risk of central nervous system damage. The QPlogHERG of emodin and rhein was within the specified range, indicating that the risk of cardiotoxicity of emodin and rhein was low. The toxicity level of emodin and rhein of Class V was relatively safe; the LD50 of quercetin was 159 mg/kg and belonged to Class III, indicating quercetin has potential safety risks. These suggested that emodin and rhein were the most promising compounds.

**TABLE 4:** Pharmacokinetic properties of the key compounds.

Compound	RO5^a^	%HOA^b^	CNS^c^	QPlogBB^d^	QPlogHERG^e^	LD50 (mg/kg)	Toxicity class^f^	Hepatotoxicity
Emodin	0	68.29	−2	−1.53	−4.32	5,000	V	Inactive
Rhein	0	47.47	−2	−1.97	−2.70	5,000	V	Inactive
Quercetin	0	52.20	−2	−2.40	−5.13	159	III	Inactive

a Note: acceptable range: Max.4.

b acceptable range: 0–100.

c acceptable range: −2 (inactive) +2 (active).

d acceptable range: −3.0 to 1.2.

e acceptable range: <−5.

f [Class I: death after swallowing (LD50 ≤ 5); Class II: death after swallowing (5 < LD50 ≤ 50); Class III: toxic after swallowing (50 < LD50 ≤ 300); Class IV: harmful after swallowing (300 < LD50 ≤ 2000); Class V: may be harmful after swallowing (2000 < LD50 ≤ 5,000); Class VI: non-toxic (LD50 > 5,000)].

## 6 Conclusion

Based on TCM theory and documentation, HSBDF including two classical prescriptions of MXSGD (*E. sinica*, *S. armeniacae amarum*, and *Glycyrrhiza uralensis*) and HXZQP (*A. rugosus*, *M. officinalis*, *R. Pinelliae Preparata*, *A. lancea*, *P. cocos*, and *Glycyrrhiza uralensis*) was recommended to treat ALI at the later stages of COVID-19 by the NHC. Molecular docking results showed that the total number of compounds with potential inhibitory activities of MXSGD (120) against IL-6R and TNF-α was higher than that of HXZQP (110). The results indicated that MXSGD was more outstanding in anti-inflammation than HXZQP, which up to the TCM theory that MXSGD can regulate lung mechanisms such as diffuse the lung qi. Also, previous studies confirmed that MXSGD played a role in suppressing cytokine storm and immune regulation and protecting the pulmonary alveolar–capillary barrier (Wang et al., 2020).

In terms of single herb, we found that *Glycyrrhiza uralensis*, *E. sinica*, and *R. officinale* were key herbs with the potential to inhibit inflammation, which was of great significance to ameliorate ALI. These results are in accordance with the Chinese medicine theory that *Glycyrrhiza uralensis* and *E. sinica* belong to the lung channel; *R. officinale* belongs to the interior–exterior of the large intestine channel and the lung. Therefore, *Glycyrrhiza uralensis*, *E. sinica*, and *R. officinale* had properties to treat lung-related diseases. These results were a significant guide for the compatibility of TCM in the treatment of ALI.

Quercetin was outstanding in inhibiting anti-inflammation and anti-oxidative stress in this study. Considering that quercetin has potential safety risks, the structural optimization of quercetin to avoid toxicity but retain anti-inflammatory and anti-oxidant features is worth further study.

In summary, this study made good use of chemical bioinformatics analysis, including molecular docking, LC/MS, network pharmacology, test *in vitro*, molecular dynamic simulation, binding interaction pattern analysis, and pharmacokinetic properties to systematically study the regulatory mechanisms of HSBDF and its active compounds in plasma in treating ALI. Overwhelming lung inflammation, oxidative stress, and cell apoptosis were involved in the whole pathological process of ALI. Our results suggested that HSBDF and its active components in plasma (quercetin, emodin, and rhein) have significant effects on anti-inflammation, anti-oxidative stress, and decreasing cell apoptosis in treating ALI. Moreover, quercetin, emodin, and rhein, key active components in plasma, had a stable affinity with IL-6R and TNF-α, which are worthy of further development and utilization for ALI.

## Data Availability

The original contributions presented in the study are included in the article/Supplementary Material, further inquiries can be directed to the corresponding authors.

## Supplementary Material

Supplementary Figure S1 Cell viability for MLE12 after treatments of HSBDF and key compounds in plasma (cell viability was detected by CCK-8,**P* < 0.05 versus blank).

Supplementary Figure S2 Histogram and percentage graph of protein–active compound interaction in molecular dynamics simulation. **(B,C)** Protein–ligand interaction of rhein and quercetin with IL-6R; **(C,D)** protein–ligand interaction of rhein and emodin with TNF-α.
