# Supplementary figures and images for: Deciphering the Active Compounds and Mechanisms of HSBDF for Treating ALI via Integrating Chemical Bioinformatics Analysis
Source: Front Pharmacol. 2022 Jun 2;13:879268. doi: 10.3389/fphar.2022.879268 (PMC9201258; doi:10.3389/fphar.2022.879268)

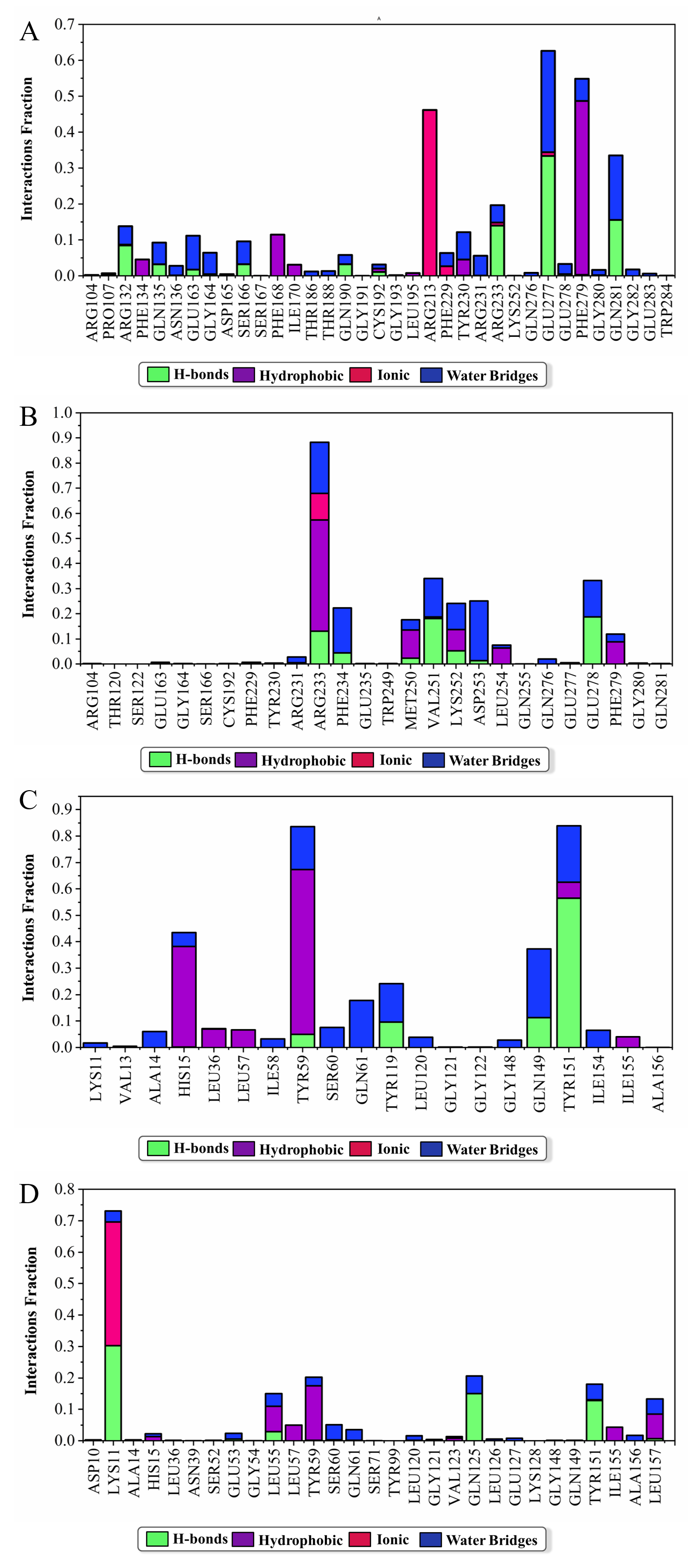

Supplement: Supplementary file 1 [file Image2.TIF]
